# Immunogenic Subviral Particles Displaying Domain III of Dengue 2 Envelope Protein Vectored by Measles Virus

**DOI:** 10.3390/vaccines3030503

**Published:** 2015-07-03

**Authors:** Indira S. Harahap-Carrillo, Ivonne Ceballos-Olvera, Jorge Reyes-del Valle

**Affiliations:** School of Life Sciences, Arizona State University, Tempe, AZ 85281, USA; E-Mails: iharahap@asu.edu (I.S.H.C.); Ceballos-Olvera@asu.edu (I.C.O.)

**Keywords:** measles vectors, HBsAg subviral particles, dengue vaccine platform

## Abstract

Vaccines against dengue virus (DV) are commercially nonexistent. A subunit vaccination strategy may be of value, especially if a safe viral vector acts as biologically active adjuvant. In this paper, we focus on an immunoglobulin-like, independently folded domain III (DIII) from DV 2 envelope protein (E), which contains epitopes that elicits highly specific neutralizing antibodies. We modified the hepatitis B small surface antigen (HBsAg, S) in order to display DV 2 DIII on a virus-like particle (VLP), thus generating the hybrid antigen DIII-S. Two varieties of measles virus (MV) vectors were developed to express DIII-S. The first expresses the hybrid antigen from an additional transcription unit (ATU) and the second additionally expresses HBsAg from a separate ATU. We found that this second MV vectoring the hybrid VLPs displaying DIII-S on an unmodified HBsAg scaffold were immunogenic in MV-susceptible mice (HuCD46Ge-IFNar^ko^), eliciting robust neutralizing responses (averages) against MV (1:1280 NT_90_), hepatitis B virus (787 mIU/mL), and DV2 (1:160 NT_50_) in all of the tested animals. Conversely, the MV vector expressing only DIII-S induced immunity against MV alone. In summary, DV2 neutralizing responses can be generated by displaying E DIII on a scaffold of HBsAg-based VLPs, vectored by MV.

## 1. Introduction 

Dengue virus (DV) is the most prevalent arthropod-borne viral disease in the world and its importance both in terms of number of people infected and economic impact for endemic countries is unrivaled among arboviruses [1]. A vaccine to prevent infection by any of the four described DV serotypes has been an elusive goal for more than six decades. The most advanced DV vaccine is based on a chimeric virus where the relevant DV serotype envelope is expressed by and encases the replication machinery of the yellow fever virus vaccine strain 17D [2]. Three extensive clinical trials of a tetravalent formulation of this vaccine have been conducted in areas of high dengue endemicity thus far, finding an unbalanced protection among DV serotypes [3,4,5]. In particular the response against DV2 is suboptimal. After three doses of the vaccine cocktail, the protection against febrile diseases caused specifically by DV2 goes from only approximately 35% to 50%, which suggest the need to reformulate the vaccine or the immunization approach.

It is widely accepted that neutralizing immunity against the DV envelope (E) protein is the goal to attain with any immunization strategy [6,7,8]. On the DV surface, 180 copies of the E glycoprotein arrange in dimers parallel to the surface of the virion with icosahedral symmetry. Recently, the interface between the constitutive monomers in a dimer has been proposed as an ideal structural target for broadly neutralizing immunization [8]. Nevertheless, in this paper we focus on a different thermodynamically stable region of self-folding capability, proposed to be responsible for interaction with the cellular receptor, the DV E domain III (DIII) [9,10,11]. DV DIII (around 100 amino acids in length) bulges slightly on the smooth surface of mature virions and forms the topographic epicenter for the five- and three-fold axes of virion symmetry. Antibodies against DIII have the potential to be highly neutralizing and protective [12,13]. For effective vaccination, however, the small molecular size of DV EDIII requires the formulation of an efficient and immunogenic platform on which to display this neutralization determinant.

Antigenic epitopes displayed on highly structured particles are very immunogenic in terms of neutralizing immunity, which constitutes the basis for most of the vaccines for human use. As an alternative for viral attenuation in vaccine development, the use of subunit antigens made of viral envelope protomers is a proven strategy. Hepatitis B and human papilloma virus vaccines are based on empty virus-like particles (VLPs) resulting from the heterologous expression of envelope or capsid viral proteins [14,15]. Others have exploited the immunogenicity of HBsAg VLPs immunogenicity for vaccine to develop vaccines against heterologous pathogens [16,17,18,19] or for non-vaccine purposes [20,21]. Particularly relevant to this paper is the most advanced anti-malarial preventative vaccine, RTS,S. This vaccine consists of hybrid HBsAg particles expressed in yeast that incorporate an abbreviated form of the circumsporozoitic antigen fused to HBsAg (RTS). A recent publication documented that the efficiency of an immunization schedule consisting of three doses and a booster of adjuvanted RTS,S vaccine successfully averted numerous malaria cases (clinical trials NCT00866619), especially in children aged 5 to 17 months [22]. The adjuvant for this vaccine formulation (AS201, Glaxo) has proven to be important to the immunogenicity of this approach.

In this paper, we have designed a hybrid form of the hepatitis B small surface antigen (HBsAg, also known as S antigen) displaying DV2 EDIII neutralization determinants. As an expression platform and adjuvant, we used a vaccine-equivalent recombinant measles vector, MVvac2 [23,24]. We tested our vaccine in mice susceptible to measles virus (MV) to demonstrate the immunogenicity of our candidates [25,26]. All of the experimental animals tested developed protective neutralizing responses against all three viruses (MV, HBV and DV) simultaneously when vaccinated with the candidate that successfully assembled and secreted an HBsAg-based subviral particle displaying DV2 E DIII.

## 2. Experimental Section

### 2.1. Cells and Viruses

Vero/hSLAM cells [27] were maintained in Dulbecco’s modified Eagle’s medium with high glucose concentration (DMEM, Sigma-Aldrich, St. Louis, MO, USA) supplemented with 5% fetal bovine serum (FBS, Atlanta Biologicals, Flowery Branch, GA, USA), 1% penicillin–streptomycin (Sigma-Aldrich) and 0.5 mg/mL G418 (Enzo Life Sciences, Farmingdale, NY, USA) to maintain hSLAM expression selection. Helper 293-3-46 cells [28] a derivative from HEK 293 cells were maintained in DMEM with 10% FBS, 1% PS, and 1.2 mg/mL G418.

Recombinant MVs were rescued using the method of Radecke *et al.* [28] modified by Parks *et al.* [29]. Briefly, helper 293-3-46 cells were transfected with 10 μg of the relevant measles full-length plasmid and a 20 ng of a plasmid expressing MV polymerase (pEMCLa) and then co-cultured with Vero/hSLAM 48 h after transfection. After detecting cytopathic effect in mixed cultures, individual syncytia were transferred to and propagated in Vero/hSLAM cells. To prepare stocks of the viral clones thus generated, Vero/hSLAM cells were infected at a multiplicity of infection (MOI) of 0.03 and incubated at 37 °C. When approximately 80% cytopathic effect was observed, cells were scraped in Opti-MEM (Life Technologies, Grand Island, NY, USA) and viral particles were released by two freeze–thaw cycles. For MVvac2 (DIII-S, S)P vector, viral titer was increased by enhancing the number of infected cells three times and reducing the volume in which the virus was collected.

Multi-step growth kinetics of the recombinant vectors were measured by infecting 10^5^ Vero/hSLAM cells at an MOI of 0.03 in a six-well plate and incubating them at 37 °C. Infected cells were collected and lysed by a single freeze-thaw cycle at prescribed times post-infection, and the 50% tissue culture infectious dose (TCID_50_) was assessed in Vero/hSLAM using the Spearman-Kärber end-point dilution method [30].

### 2.2. Construction and Recovery of Recombinant MVs

**Figure 1:**
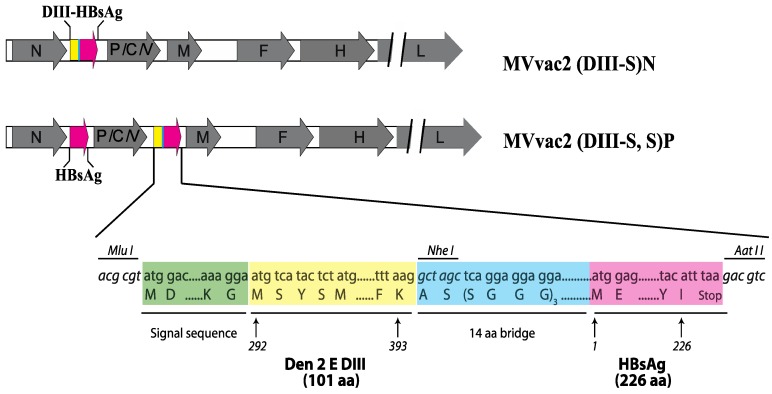
Schematic representation of recombinant measles vectors expressing hybrid HBsAg glycoprotein antigens. Gray arrows represent the MV cistrons, while colored arrows represent the HBsAg or its recombinant hybrid derivate coding sequences. In the lower panel, an abbreviation of the inserted sequence is shown (colored boxes with aa abbreviations are not drawn to scale); important restriction sites (italics) and the relevant amino acid sequence and positions for the signal sequence of the immunoglobulin light chain, Dengue 2 Envelope glycoprotein domain III (aa 292–393), the protein bridge and HBsAg (aa 1–226) sequence are shown.

To generate the artificial, hybrid DIII-S antigen coding sequence, we used splicing overlapping PCR (see lower panel of Figure 1). First, we amplified the corresponding E DIII region from DV2 (strain 16681) by RT-PCR. This amplicon is flanked at the 5' end by an MluI site and the coding sequence of the light immunoglobulin chain signal peptide, and by the coding sequence of a 15 amino acid bridge at the 3' end. Then, we amplified by PCR the coding sequence of HBsAg from pB(+)MVvac2(HBsAg)N. This amplicon was flanked by the complementary region of the afore-mentioned 14-amino acid bridge at the 5' end and by an AatII restriction site at the 3' end. Both fragments were spliced together by PCR and the resulting product cloned into the shuttle vector pJET1.2. The hybrid gene and gene boundaries were sequenced (PCR primer sequences available upon request) To generate the plasmid for rescue of MVvac2(DIII-S)N, we used the backbone provided by pB(+)MVvac2(HBsAg)N [24]. The MV genome coding capacity in this plasmid is identical to those of the Moraten/Schwartz vaccine strains [23] containing an additional transcription unit (ATU) inserted downstream of the nucleocapsid (N) cistron that directs the expression of the foreign gene. Following MluI and AatII enzymatic digestion, the hybrid DIII-S coding sequence was swapped for the HBsAg insert. To generate the plasmid pB(+)MVvac2(DIII-S,S)P, used to rescue the corresponding recombinant vector, we cloned the hybrid DIII-S antigen coding sequence in a locus downstream the phosphoprotein gene (P) using MluI and AatII sites and the plasmid MVvac2(HBsAg)P [23]. Then, the restriction fragment SfiI-SacII from pB(+)MVvac2(HBsAg)N containing the N coding sequence and HBsAg as an ATU downstream of it, was interchanged for the corresponding fragment in the pB(+)MVvac2(DIII-S)P vector, thus generating pB(+)MVvac2(DIII-S,S)P, with HBsAg encoded in an ATU downstream of MV N and DIII-S encoded in an ATU downstream of MV P. For all of the constructed full-length plasmids we corroborated that the total number of nucleotides comprising the recombinant viral vector was divisible by six to ensure efficient replication, as reported [31].

### 2.3. Protein Expression Analysis

For protein expression analysis, 1 × 10^6^ Vero/hSLAM cells were seeded in 100-mm-diameter dishes and infected at an MOI of 0.3 with the recombinant virus or were mock-infected. Forty hours after infection, cells were washed three times with phosphate-buffered saline (PBS) then lysed with RSB-NP40 buffer (1.5 mM MgCl_2_, 10 mM Tris-HCl, 10 mM NaCl, and 1% Nonidet P-40, Sigma-Aldrich) plus protease inhibitors (cOmplete Mini Protease Inhibitor Tablets, Roche Diagnostics, Manheim, Germany). The protein extracts were mixed with Laemmli buffer alone (Bio-Rad Laboratories, Hercules, CA, USA) for HBsAg detection, or with Laemmli buffer containing β-mercaptoethanol for DV2 E DIII detection, and denatured at 96 °C for 10 min. Cell lysates were separated by SDS polyacrylamide gel electrophoresis (PAGE) in a 10% acrylamide gel. Following electrophoretic separation, proteins were transferred to nitrocellulose for immunoblotting using a 1:1000 dilution of rabbit polyclonal anti-HBsAg antibody coupled to HRP (OBT0990P, AbD Serotec, Raleigh, NC, USA), and a 1:500 dilution of a mouse monoclonal antibody directed against DV2 E DIII (GTX29202, GeneTex, Irvine, CA, USA). The signal was developed after incubation with a horseradish peroxidase (HRP)-conjugated secondary antibody (GE Healthcare, Little Chalfont, UK) when appropriate, using a chemiluminescence kit (SuperSignal West Pico Chemiluminescent Substrate, Pierce Biotechnology, Rockford, IL, USA).

### 2.4. Viral Particles Purification

To purify released particles, supernatants of 5 × 10^6^ cells infected with the parental strain MVvac2, MVvac2(HBsAg)N, MVvac2(DIII-S)N, or MVvac2(DIII-S,S)P were initially clarified by centrifugation at 4635 rpm for 15 min in an SX4750 rotor. The clarified supernatants were pelleted by ultracentrifugation at 45,000 rpm for18 h in an SW 28 rotor. Pellets were re-suspended in TNE buffer (10 mM Tris (pH 7.8), 100 mM NaCl, 1 mM EDTA) containing 20% sucrose, then overlaid in a 30% to 60% discontinuous sucrose gradient. Fractions were ultracentrifuged to equilibrium at 37,000 RPM in a MLS50 rotor. Fractions from top to bottom were weighed and their density calculated. A 25 μL aliquot was analyzed by Western blot as described before.

### 2.5. Mouse Inoculations

The Arizona State University Institutional Animal Care and Use Committee sanctioned all experimental procedures. To determine the immunogenicity of recombinant viruses in a MV-susceptible model [25], groups of three to seven HuCD46Ge-IFNar^ko^ mice were inoculated initially by the intraperitoneal (i.p.) route with one or two doses of 10^5^ TCID_50_ of the relevant recombinant MV vector. Four weeks after the initial inoculation or two weeks after the booster dose, the animals were humanely euthanized and exsanguinated. Serum was separated and heat-inactivated at 56 °C for 1 h. Heat-inactivated serum aliquots were kept frozen at −30 °C until analyzed.

### 2.6. Analysis of Immunogenicity

To determine MV neutralization titer, serial two-fold dilutions of heat-inactivated sera in Opti-MEM were incubated with 100 TCID_50_ of MVvac2 for 1 h at 37 °C. Vero/hSLAM cells were added to the serum–virus mixtures and the plates were then incubated at 37 °C for three days, at which time neutralization titer was assessed as the highest dilution of serum capable of complete neutralization of infectivity, defined as the absence of MV cytopathic effect. Neutralization titers were reported as the average of a determination made in triplicate.

To determine DV2 neutralization titer, 10^5^ DV2 PFU were incubated with heat-inactivated serum diluted 1:5 in Opti-MEM in a 200 µL volume for 1 h at 37 °C. Infectivity after serum incubation was determined using a DV2 plaque assay in BHK-21 cells. Briefly, ten-fold serial dilutions of the virus/serum mixtures were generated using Opti-MEM and added to 2.4 × 10^6^ BHK-21 cells in a 24-well plate and then incubated for 4 h at 37 °C. At this time, 500 µL of 3% carboxymethyl cellulose was added to each well and plates were incubated for 5 days at 37 °C. The wells were then washed, the monolayer was fixed and stained with acetic acid/naphtol blue-black and plaques were counted. The neutralization index was expressed as the logarithmic difference between the averaged DV2 titers when incubated with control, namely, that of animals inoculated with MVvac2, MVvac2(HBsAg)N and preimmune, and DV2 titers when incubated with sera of animals from the relevant experimental group. Neutralization indexes thus determined were graphed as Log_10_NI. In another approach, we performed a standard 50% plaque reduction neutralization assay (PRNT_50_) using pooled sera from each experimental group. Briefly, 50 DV2 PFU were incubated with two-fold serum dilutions (from 1:5 to 1:320) for 1 h at 37 °C. Remaining infectivity was determined by plaque assay, as described above. The neutralization titer was defined as the dilution that resulted in a 50% reduction in plaque numbers, as compared to unrelated serum.

Anti-HBsAg antibody titers were determined with a species-independent quantitative ELISA kit (Alpha Diagnostic Intl. Inc., San Antonio, TX, USA) and expressed as milli-international units per milliliter by comparison with World Health Organization standards supplied by the manufacturer.

## 3. Results and Discussion

### 3.1. Design of Recombinant MVs Vectoring Hybrid s Secreting Hybrid DIII-HBsAg Forms

To induce the assembly and secretion of particulate antigenic material displaying neutralizing epitopes from DV2, we took advantage of the robust expression platform of MV. MVvac2 is a recombinant version of the attenuated strains Moraten and Schwarz, currently used in the measles eradication effort. Its genome has the same coding capacity as these widely used vaccine strains. Initially, we designed a hybrid form of the hepatitis B small surface antigen (HBsAg, also called S antigen), composed of an artificial ORF lead by the light immunoglobulin chain signal sequence fused in frame with amino acids 292 to 393 from the DV2 E glycoprotein protein linked to the amino terminus of the HBsAg (ady type). DV2 E DIII, corresponding to the afore-mentioned amino acids of E, has an independent, immunoglobulin-like folding dependent on a conserved disulfide bridge. To promote independent folding by the DV2 E DIII and HBsAg modules we separated them by a 14 amino acid bridge with a predicted α-helical structure. The coding sequence of this artificial, hybrid DIII-HBsAg (DIII-S) was inserted as an additional transcription unit (ATU) downstream the MV nucleocapsid (N) gene to generate the full length plasmid pB(+)MVvac2(DIII-S)N. In MV vectors, as in most of the *Mononegavirales*, the transcription of a determined cistron depends on its position relative to the 3' end of the genome [32,33]. Cistrons proximal to the 3′ leader are transcribed more intensively due to gradual attenuation at gene boundaries. This background allows us to predict the relative expression of vectored foreign antigens. To generate a MV vector where unmodified HBsAg can be used as scaffold to incorporate the hybrid antigen, we followed the example of other expression platforms where a three-fold higher ratio of HBsAg is used as a scaffold to incorporate a hybrid version of itself into VLPs [16]. We inserted our hybrid DIII-S antigen downstream of the MV P gene in a construction that expresses the native HBsAg from an ATU downstream of the N position to create the full-length plasmid pB(+)MVvac2 (DIII-S,S)P. Previously reported full-length constructions pB(+)MVvac2, the parental strain, and pB(+)MVvac2(HBsAg)N, a derivative plasmid encoding for the HBsAg from downstream of N, were used as controls. We recovered the corresponding viruses by established reverse genetic techniques.

### 3.2. Replication Profile of Recombinant MV Vectors

**Figure 2:**
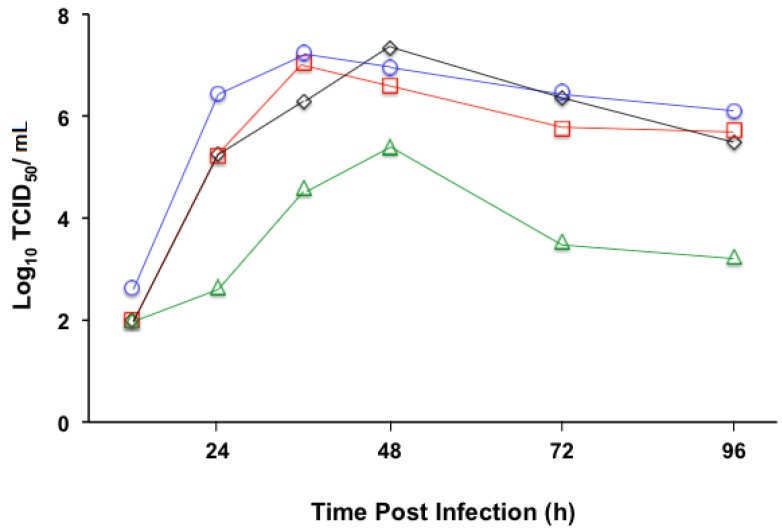
Time course of cell-associated MV production in Vero/hSLAM cells infected with four recombinant viruses: MVvac2 (diamonds, black line), MVvac2(HBsAg)N (squares, red line), MVvac2 (DIII-S)N (circles, blue line), and MVvac2(DIII-S,S)P (triangles, green line). Viral titers (expressed as the log_10_ TCID_50_/mL) indicated on the vertical axes were measured 12, 24, 36, 48, 72, or 96 h post infection. Averages of three independent experiments conducted separately are indicated. For clarity, standard deviations of the mean are not shown.

Once the recombinant viruses were recovered and viral preparations produced, we started characterizing their potential to be used as vaccine vectors based on replication efficiency. To document the replication fitness of MVvac2(DIII-S)N and Mvac2(DIII-S,S)P *in vitro*, multistep growth kinetic analyses were performed. We used Vero/hSLAM cells as hosts and infected them with a multiplicity of infection of 0.03. Cell-associated viral progenies were collected at different time points and MV infectivity was determined as TCID_50_. As shown in Figure 2, we observed two viral replication profiles. While MVvac2(DIII-S)N followed a replication efficiency that closely resembled that of the parental strain MVvac2 or the reference strain MVvac2(HBsAg)N, with maximum titers of around 10^7^ TCID_50_/mL at 48 h, Mvac2(DIII-S,S)P reached a maximum titer approximately 22 to 50 times lower of 10^5.15^ TCID_50_/mL at 48 h post infection. Thus, the simultaneous insertion of two ATUs had a measurable effect upon the replication of Mvac2(DIII-S,S)P. This negative effect on replication has been observed in other MV vectors with multiple ATUs due to transcriptional “dilution” of essential MV genes downstream of the ATU insertion [32,34]. Despite this negative effect on replication, we successfully prepared viral stocks with a titer of 10^6^ TCID_50_/mL.

### 3.3. Expression of Hybrid DIII-HBsAg Antigens by MV Vectored Vaccines

**Figure 3:**
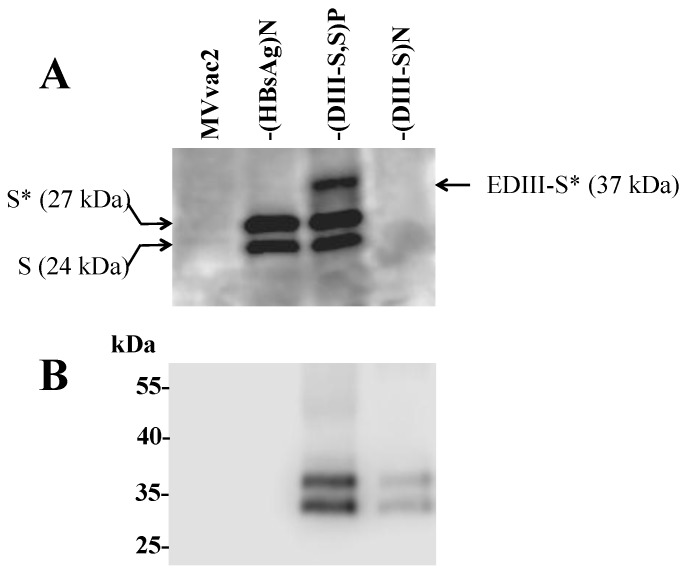
Analysis of expression of the hybrid DIII-HBsAg in lysates from recombinant MV-infected cells (indicated on the top of each panel). In (**A**) SDS-PAGE under non-reducing conditions using a monoclonal antibody against HBsAg (the S antigen); the molecular weight of the two forms of S are shown to the left and the molecular weight of the hybrid DIII-S to the right. The asterisk indicates glycosylated forms. In (**B**) SDS-PAGE under reducing conditions using a monoclonal antibody against dengue envelope protein domain III.

We demonstrated the expression of hybrid DIII-S forms by our recombinant vectors through immunoblot of infected Vero/hSLAM cell lysates using specific monoclonal antibodies (Figure 3). Cells were infected at a MOI of 0.5 with MVvac2, MVvac2(HBsAg)N, or our two engineered vectors, MVvac2(DIII-S, S)P and MVvac2(DIII-S)N, and cells were lysed 24 h post infection. Since the reactivity of most anti-HBsAg antibodies depends upon the integrity of the “a” epitope, which is preserved by a conserved disulfide bridge, the anti-HBsAg Western blot presented in Figure 3A was performed under non-reducing conditions. As expected, a doublet of 27 and 24 kDa (theoretical molecular weight) was observed in cell extracts obtained from MVvac2 (HBsAg)N infected cells corresponding to the glycosylated and non-glycosylated isoforms of HBsAg. In the MVvac2 (DIII-S, S)P infected cell extracts lane we observed, in addition to the expected HBsAg doublet, a band corresponding to the glycosylated form of the hybrid DIII-S antigen (with a theoretical molecular weight of 37 kDa). It is possible that the non-glycosylated form of the hybrid antigen was not visible due to the non-reducing conditions of the electrophoresis and the polyacrylamide concentration (10%). Conversely, in the MVvac2 (DIII-S)N infected cell extract we were not able to detect any protein band recognized by the anti-HBsAg antibody. As mentioned, recognition by our anti-HBsAg monoclonal antibody requires conservation of the semi-native structure of its epitope provided by the non-reducing conditions. We speculate that the proper folding of the hepatitis B S antigen portion in hybrid DIII-HBsAg, when expressed individually, is negatively impacted by the lack of *in cis* HBsAg expression, as is provided in the MVvac2 (DIII-S, S)P vector: in other words, unmodified HBsAg may promote the proper folding of hybrid DIII-HBsAg.

To document that the antigenic material was also vectoring DV2 epitopes present in domain III, a Western blot was developed with specific antibodies against DV2 Domain III. Since this antibody is directed against a lateral region of domain III in a linear epitope, the electrophoresis was carried under reducing conditions. Figure 3B documents, for both of our engineered vectors, recognition by this antibody of two protein bands with an electrophoretic migration of 37 and 34 kDa, corresponding to the glycosylation isoform of the hybrid DIII-HBsAg. The signal observed in cells infected with MVvac2(DIII-S)N is comparatively lower than that of MVvac2 (DIII-S, S)P infected cells supporting the notion that the hybrid antigen expression is impaired when no unmodified HBsAg is expressed *in cis*. All of the infected cells extracts were positive for the presence of MV N detected by a polyclonal anti-N serum (data not shown).

### 3.4. Secretion of Hybrid Virus-Like Particles with an HBsAg-Like Density

**Figure 4:**
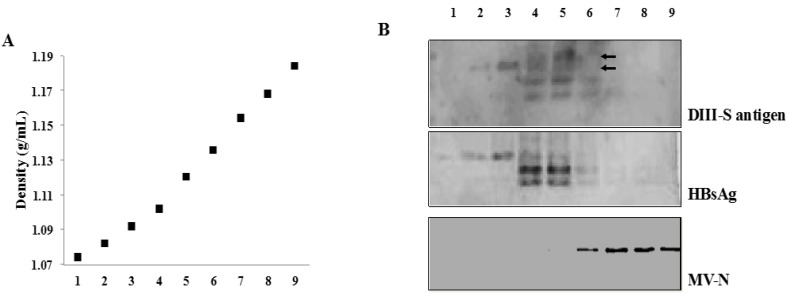
(**A**) Materials released from Vero/hSLAM cells infected with MVvac2DIII-S,S)P or MVvac2(HBsAg)N collected 72 h after infection were clarified, and particulate material pelleted, loaded on a 20% to 60% sucrose gradient, and centrifuged to equilibrium. Five hundred microliter fractions were collected from the top (left) to the bottom (right) and weighed. The dotted line shows a representative density profile. (**B**) Aliquots of each fraction were separated by 12.5% SDS-polyacrylamide gel electrophoresis, immunoblotted, and probed with anti-HBsAg antibodies for fractions coming from cells infected with MVvac2DIII-S,S)P (upper panel) or MVvac2(HBsAg)N (medium panel). The lower panel indicates the migration of infectious MV virions.

The immunogenicity of the only two viral vaccines approved for human use based on a subunit approach derives from their VLP formulation. This particulate material represents a powerful antigenic stimulus due to its ability to display repetitive antigenic determinants. In consequence, we aimed to test for the presence of hybrid VLPs in supernatants of MVvac2(DIII-S, S)P and MVvac2(DIII-S)N infected cells fractionated by a discontinuous sucrose gradient. For this experiment we used supernatants from MVvac2(HBsAg)N-infected cells prepared in parallel as a positive control because their capability to secrete VLPs with a native sucrose density has been previously documented [23,35]. As shown in Figure 4, we detected the presence of the hybrid DIII-S antigen in MVvac2(DIII-S, S)P-infected cells supernatants with a similar sucrose density (1.10–1.12 g/L, fractions 4 and 5) to that of the native HBsAg secreted into the supernatants of MVvac2(HBsAg)N infected cells. As expected, MV viral particles migrated in more dense fractions, supporting the notion that the hybrid antigen is not modifying the surface of the MV virion but instead is secreted as an independent particle. Due to the lack of reactivity against the anti-HBsAg antibody (Figure 3), performing a similar analysis with supernatants from MVvac2(DIII-S)N infected cells would not be fruitful. We reasoned that its reactivity to anti-dengue 2 antibodies would assist us in its detection in the corresponding fractions if particulate material were secreted, but we were not able to detect the presence of the hybrid DIII-S antigen in supernatants of MVvac2(DIII-S)N infected cells using this antibody. In sum, we were able to document the incorporation of hybrid DIII-S antigen on a scaffold of HBsAg particles without affecting its sucrose density.

### 3.5. Immunogenicity of Recombinant MVs Vectoring Hybrid DIII-HBsAg Forms

To document the immunogenicity of our MV vectors carrying antigenic determinants from three different viruses, we vaccinated groups of HuCD46Ge-IFNar^k^° genetically modified mice. This host expresses one of MV receptors (CD46) with human-like distribution in a type I interferon inactivated background to allow MV transient replication and is the gold standard small animal model for measles vaccine immunogenicity [25]. Groups of three to seven animals received one or two 10^5^ TCID_50_ intraperitoneal doses (indicated by X1 or X2 in Table 1 and Table 2) of the parental strain MVvac2, the reference strain MVvac2(HBsAg)N, or the experimental recombinant vectors MVvac2(DIII-S, S)P or MVvac2(DIII-S)N. We did not observe any signs of toxicity or illness among immunized animals. Twenty-eight days after a single dose or 14 days after a booster dose received 28 days after the primary dose, the animals were euthanized and exsanguinated; sera were isolated and de-complemented. To document measles immunogenicity (Table 1) we performed a microneutralization assay where the neutralization titer represents a 90% inhibition of MV cytopathic effect in Vero/hSLAM cells. As documented in Table 1, the animals of all experimental groups responded to the antigenic stimulus presented by the viral vector backbone. After a single dose of either MVvac2 or MVvac2(DIII-S, S)P we determined anti-MV neutralization titers of 1:1000 and 1:640 on average, respectively. It is possible that the comparatively reduced viral fitness observed *in vitro* as a result of the insertion of two ATUs (Figure 2) is responsible for the difference in immunogenicity between MVvac2(DIII-S, S)P and the parental strain. We also verified the effect of a booster dose administered 28 days after priming. Groups of animals vaccinated with MVvac2(HBsAg)N, MVvac2 (DIII-S,S)P or MVvac2(DIII-S)N had anti-MV neutralizing titers ranging from 1:1500 to 1:2560 following the boost. To document the reactivity of immune sera against DV2, we performed neutralization experiments using two related approaches (Table 2). Initially we determined the effect of a 1:5 serum dilution upon the infectivity of a 10^5^ PFU/mL DV2 viral prep. The reduction of DV2 infectivity caused by immune sera reactivity was expressed as the logarithmic neutralization index (Log_10_NI), where samples coming from animals immunized with control viruses (MVvac2 and MVvac2(HBsAg)N) set the zero neutralization for this experiment. The only experimental group that presented a consistent and robust reactivity against DV2 was the one vaccinated with MVvac2(DIII-S,S)P. After a single dose, this group’s average was 0.7 LNI (a reduction of five times in DV2 infectivity), while a second dose elicited a patent boosting effect resulting in a protective average of 1.3 LNI, a reduction of almost 20 times in DV2 infectivity. For reference, for yellow fever virus, a related arthropod borne flavivirus with similar antigenic structure, a LNI of 0.7 is considered the correlate of protection [36]. Conversely, two doses of MVvac2(DIII-S)N induced a measurable but marginal effect of 0.3 LNI on average (a reduction of two times in DV2 infectivity).

**Table 1:** Anti-Measles immunogenicity (PRNT_90_ titer).

MVvac2 X 1 ^a^		-(HBsAg)N X 2 ^b^		-(DIII-S, S)P X 1 ^a^		-(DIII-S, S)P X 2 ^b^		-(DIII-S)N X 2 ^b^	
Pre	Post	Pre	Post	Pre	Post	Pre	Post	Pre	Post
<1:10	1:1280	<1:10	>1:2560	<1:10	1:1066	<1:10	>1:2560	<1:10	>1:2560
<1:10	1:1280	<1:10	>1:2560	<1:10	1:640	<1:10	>1:2560	<1:10	1:1280
<1:10	1:1280	<1:10	>1:2560	<1:10	1:320	<1:10	1:1280	<1:10	1:1066
<1:10	1:640			<1:10	1:240	<1:10	1:1280		
<1:10	1:640					<1:10	1:640		
						<1:10	1:640		
						<1:10	1:640		

a Animals were inoculated with a single 10^5^ TCID_50_ intraperitoneal dose of the indicated virus, MV immunity was assayed 28 days post-immunization; b Animals were inoculated with two 10^5^ TCID_50_ intraperitoneal doses in a 28 days time interval of the indicated virus, MV immunity was assayed 14 days post booster dose.

**Table 2:** Anti Dengue 2 immunogenicity.

(DIII-S, S)P X 1		(DIII-S, S)P X 2		(DIII-S)N X 2	
LNI ^a^	PRNT50 ^b^	LNI	PRNT50	LNI	PRNT50
0.64	1:10	1.41	1:160	0.21	1:20
0.81		1.51		0.33	
0.64		1.51		0.21	
0.51		1.81			
		1.81			
		0.51			
		0.41			

Individual LNI titers are presented in the same order as for Table 1. a Neutralizing titer expressed as the logarithmic neutralization index (LNI), where samples coming from MVvac2 and MVvac2(HBsAg)N immune animals (eight samples) set the zero for neutralization of DV2 infectivity (see text); b PRNT_50_ obtained in pooled serum samples for each experimental group, all of tested pre-immune samples lacked reactivity against DV2, PRNT < 1:10, LNI ~ 0.

We additionally determined DV2 reactivity through a classic 50% plaque reduction neutralization assay (PRNT_50_) in pools of sera for each experimental group, where two-fold serum dilutions were incubated with 50 PFU of DV2 and the neutralization titer was quantified as the serum dilution with one half of the plaques observed in wells incubated with control serum. We documented that only animals receiving two doses of MVvac2 (DIII-S,S)P developed a robust neutralization titer (1:160). The difference in PRNT_50_ between animals vaccinated with a single MVvac2(DIII-S,S)P dose (1:10) and animals receiving two doses of MVvac2(DIII-S)N (1:20) is not significant (single two-fold dilution difference), probably resulting from experimental background. Finally, to determine the anti-HBsAg reactivity, we used a commercially available ELISA kit comparing the readings of sera from immunized animals to a standard curve constructed with an international reference. A serum pool from animals vaccinated with two doses of the reference strain MVvac2(HBsAg)N had a titer of 648 mIU/mL, while animals receiving two doses of MVvac2 (DIII-S, S)P had a titer of 787 mIU/mL. The anti-HBsAg titers level that is considered protective is >10 mIU/mL. Thus, the documented immunity against hepatitis B virus is significant. We were not able to detect any anti-HBsAg reactivity in the pooled serum sample from animals that received MVvac2 (DIII-S)N or, as expected, MVvac2. 

In summary, we have documented the capability of hybrid DV2 E DIII-HBsAg VLP particles expressed by a MV vector to elicit robust neutralizing immune responses against Dengue 2 virus and in addition, to hepatitis B and measles viruses. It is noteworthy that antibody responses against hepatitis B and measles are above protective levels, for measles 1:120mIU/mL (approximately 1:80), for hepatitis B >10 mIU/mL. The dosage and vaccination schedule of our approach is advantageous compared to other hybrid HBsAg-based vaccine platforms based on yeast or other expression platforms, where relatively large and repetitive doses are required to generate a significant immune response. Our platform also offers practical advantages with other immunization strategies based on flaviviruses E DIII vectored by measles virus [37]. In this paper, DV1 E DIII vectored by MV was not able of inducing neutralizing immune responses by itself after two IP doses using the same animal host. Interestingly, a robust immunity (1:320), similar to ours (1:160) was obtained using an MV vector expressing E DIII displayed by the ectodomain of the homologous DV membrane protein. In consequence, we reason that enhancing the expression and incorporation of the DIII-S hybrid antigen by our vector will increase the anti-DV2 immunity. This goal is worth pursuing considering that, as a reference, DV2 neutralizing immunity in non-human primates after immunization with a single 10^5^ PFU monovalent dose of Chimerivax-DV2 vaccine was, on average, 1:380 [38]. In our hands, and apparently also for others [16], the incorporation of unmodified HBsAg into the hybrid VLPs is essential for the formation of the particle and vaccine immunogenicity altogether. The ability to modulate the expression capabilities of MV vectors by altering the locus of foreign gene insertion could enhance the incorporation of hybrid HBsAg molecules into HBsAg particles. Alternatively, modifying our strategy to express the hybrid VLPs two components by a bicistronic single ATU may optimize *in vivo* vector replication fitness and vaccine immunogenicity. Furthermore, the concept of a “live” adjuvant in the form of an attenuated MV vector makes our strategy appealing and applicable. Immunogenic E DIII from the four-dengue virus serotypes could be combined into a vaccine cocktail without increasing MV vaccine dose, declared currently to be 10^3^ to 10^4^ infectious units per dose. In this regard, we have previously shown that a pediatric dose of MV-HBV divalent vaccine remains protective against a wild type measles challenge in non-human primates [24]. A pediatric vaccination given around one year of age that elicits anti-dengue, -hepatitis B and -measles protection would be cost-efficient and safe.

## 4. Conclusions

This paper provides proof-of concept for a pediatric vaccine that would protect an individual against DENV, hepatitis B virus, and MV. Our data confirmed the production of viable VLPs displaying DV2 EDIII of DENV on a hybrid HBsAg when unmodified HBsAg is expressed *in cis* from the same MV vector. The robust immune response seen in animals that received two doses of MVvac2(DIII-S,S)P suggests that to achieve protection against measles and hepatitis B in humans, a minimum of two doses are necessary, which is currently customary for measles vaccination. Simultaneously, the robust and specific anti-dengue immunity thus elicited will build protection when combined with other vaccine platforms.
